# Supplementary material for: Soil Mineral Composition and Salinity Are the Main Factors Regulating the Bacterial Community Associated with the Roots of Coastal Sand Dune Halophytes
Source: Biology (Basel). 2022 Apr 30;11(5):695. doi: 10.3390/biology11050695 (PMC9138652; doi:10.3390/biology11050695)
Supplement: Supplementary file 1 [file biology-11-00695-s001.zip › Figure S1.pdf]

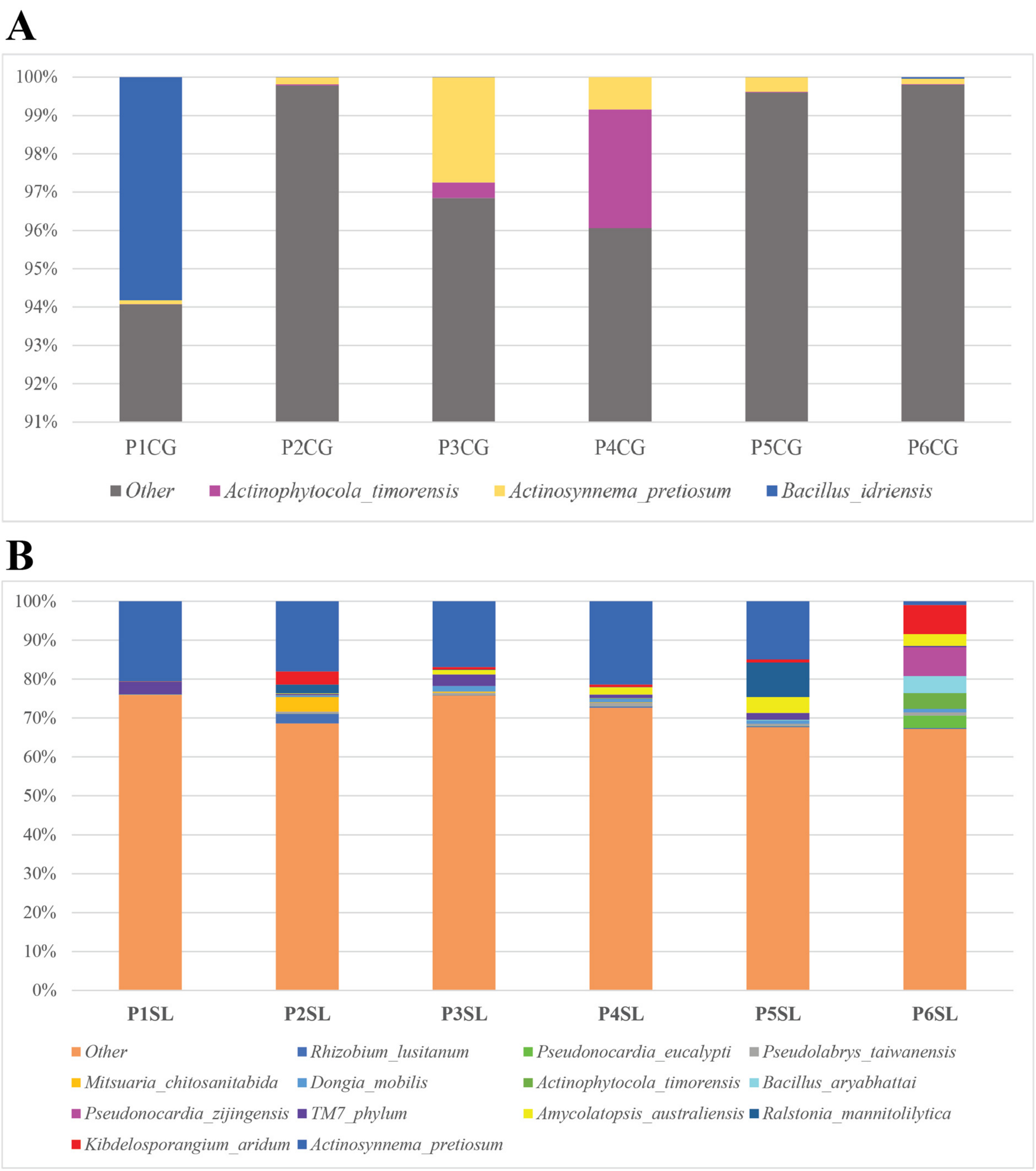


**Supplemental Figure S1**. Bacterial taxonomic composition and relative abundance at species level. A. Relative abundance of bacteria associated with the root of *Calotropis gigantea* across six different sampling populations at species level and B. Relative abundance of bacteria associated with the root of *Spinifex littoreus* across six different sampling populations at species level. Abbreviations of sampling population: P-Population, CG-*C. gigantea*, SL-*S. littoreus*.
