# Supplementary material for: Soil Mineral Composition and Salinity Are the Main Factors Regulating the Bacterial Community Associated with the Roots of Coastal Sand Dune Halophytes
Source: Biology (Basel). 2022 Apr 30;11(5):695. doi: 10.3390/biology11050695 (PMC9138652; doi:10.3390/biology11050695)
Supplement: Supplementary file 1 [file biology-11-00695-s001.zip › Table S1.pdf]

Table S1. Geographical locations of the sample collection areas.

No.	Sample	Coordinate	Environmental Setting
1.	*Spinifex littoreus* and *Calotropis gigantea* Population 1	S 08° 01.169643′, E 110°18.900061′	Parangkusumo Coastal Sand Dune, near shoreline.
2.	*Spinifex littoreus* and *Calotropis gigantea* Population 2	S 08° 01.126870′, E 110° 18.953341′	Parangkusumo Coastal Sand Dune, transition between middle zone and middle zone.
3.	*Spinifex littoreus* and *Calotropis gigantea* Population 3	S 08° 00.980402′, E 110° 19.029695′	Parangkusumo Coastal Sand Dune, middle zone.
4.	*Spinifex littoreus* and *Calotropis gigantea* Population 4	S 08° 00.868221′, E 110° 18.815405′	Parangkusumo Coastal Sand Dune, middle zone.
5.	*Spinifex littoreus* and *Calotropis gigantea* Population 5	S 08° 00.762316′, E 110° 18.836633′	Parangkusumo Coastal Sand Dune, middle zone.
6.	*Spinifex littoreus* and *Calotropis gigantea* Population 6	S 08° 00.693471′, E 110° 18.836176′	Parangkusumo Coastal Sand Dune, transition from coastal sand dune to settlement and farming area.
